# Women Doctors and the Present Crisis

**Published:** 1914-12-12

**Authors:** 


### WOMEN DOCTORS AND THE PRESENT CRISIS.

Among the probable consequences of the war it appears to be necessary to contemplate a relative failure in the supply of medical practitioners. This is, of course, a matter of great public interest, and it is not astonishing to observe that it has attracted attention in various important quarters. So far as the individual practitioner is concerned the situation is not without its redeeming features, seeing that by it his economic value and prospects are considerably improved, as the increased salaries now offered for various forms of professional work clearly show. But on general grounds no one will question that an inadequate number of medical practitioners would be greatly to the prejudice of the public, and would be especially hurtful to the poorer classes of the community. Therefore any well-advised attempt to prevent this misfortune deserves both sympathy and support.

One measure which has been publicly advocated as competent to safeguard the future is that increased numbers of women should now undertake the study of medicine; and the circumstances of the present moment are urged as a justification of this advice. It is pointed out, and quite truly, that just now there is an unusual demand for medical women to fill positions which hitherto have been held by men. This applies particularly to the resident appointments in hospitals, and perhaps also to some extent to the junior branches of the public health service, and is due to the considerable number of young medical men who have patriotically volunteered for military service. Again, it is obvious that the claim for soldiers must mean that a certain number of young men who in ordinary times would have entered our medical schools, or would have continued their studies there, have joined the Army, and that in consequence there will be during the next few years a diminished output of male practitioners. Hence it would seem that both the immediate position and the prospects of the future offer increased attractions to women to enter on the study of medicine.

It is quite true that in not a few hospitals women are being accepted as house physicians and house surgeons, and in the circumstances we are in complete sympathy with this policy. To diminish the hospital provision for the civil population by closing wards or out-patient departments merely because the governing body or the staff objects to the introduction of women resident officers seems to us quite beyond defence. At the same time it is necessary to avoid exaggerating the prospects which the medical profession offers to women. Will they survive the free competition of a full market after peace is restored? The hospital positions accessible to them to-day are largely due to the absence of this competition; they are of a subordinate order and carry no prospect of accession to the full staff; and we have certainly not heard of any medical women being called to fill vacancies caused by the absence of "our best physicians and surgeons . . . at the Front." Is it not unsafe to found the prospects of medical women on circumstances which are admittedly temporary and exceptional? The movement has had a considerable trial and has gained a certain measure of success. There seems at present little reason to believe that it will have any extraordinary expansion. Hence, for our own part, we should be loath to urge any woman to take up the study of medicine merely because at the moment, and perhaps for a year or two, there may be a relative deficiency of male practitioners. Old conditions are likely to bring back old tendencies and habits, and, if so, the law of averages will assert itself.

On this matter we have always stood for the policy of the "open door" and for the removal of all hindrances and restrictions, but many good judges are not convinced that the general welfare would be promoted by a considerable substitution of women for men in the ranks of those who serve the public as medical practitioners. If, therefore, the "shortage" of doctors is to be met in this fashion it remains to be seen if the remedy prove a satisfactory one. Hence on public as well as on personal grounds we suggest that some caution should be exercised in endorsing a suggestion that the present emergency means inevitably an assured position and income for women who will adopt a medical career.

